# Progress in enzyme-powered micro/nanomotors in diagnostics and therapeutics

**DOI:** 10.1016/j.bioactmat.2024.12.022

**Published:** 2025-01-13

**Authors:** Jinpeng Zhao, Banghui Wang, Mingzhe Yan, Yuxin Liu, Ruizhe Zhao, Xuezhe Wang, Tianyi Shao, Yifei Li, Muhammad Imran, Mingze Ji, Hong Zhao, Carlos F. Guimarães, Guotai Li, Qihui Zhou, Rui L. Reis

**Affiliations:** a Qingdao Key Laboratory of Materials for Tissue Repair and Rehabilitation, Shandong Engineering Research Center for Tissue Rehabilitation Materials and Devices, School of Rehabilitation Sciences and Engineering, University of Health and Rehabilitation Sciences, Qingdao, 266113, China; b School of Basic Medicine, Qingdao University, Qingdao, 266021, China; c Department of Biosciences, COMSATS University, Islamabad Park Road, Islamabad, Pakistan; d Department of Bone, Huangdao District Central Hospital, Qingdao, 266555, China; e 3B's Research Group-Research Institute on Biomaterials, Biodegradables and Biomimetics, University of Minho, Headquarters of the European Institute of Excellence on Tissue Engineering and Regenerative Medicine, Guimarães, 4805-017, Portugal; f ICVS/3B's – Portuguese Government Associate Laboratory, University of Minho, Braga, Guimarães, Portugal

**Keywords:** Enzyme catalysis, Micro/nanomotors, Self-propulsion, Theranostics, Biomedical applications

## Abstract

Enzyme-powered micro/nanomotors (EMNMs) represent cutting-edge research taking advantage of enzymes as biocatalysts to provide a driving force for micro/nanomotors. Up to now, EMNMs have been designed to be powered by catalase, urease, lipase, collagenase, compound enzymes, *etc*. They not only have good biocompatibility and biosafety but also possess the unique ability to utilize physiologically relevant fuel to achieve autonomous propulsion through *in vivo* catalytic reactions. This innovation has opened exciting possibilities for medical applications of EMNMs. Given the fact that the human body is naturally abundant with substrates available for enzymatic reactions, EMNMs can effectively exploit the complex microenvironment associated with diseases, enabling the diagnosis and treatment of various medical conditions. In this review, we first introduce different kinds of EMNMs applied in specific environments for the diagnosis and treatment of diseases, while highlighting their advancements for revolutionizing healthcare practices. Then, we address the challenges faced in this rapidly evolving field, and at last, the potential future development directions are discussed. As the potential of EMNMs becomes increasingly evident, continued research and exploration are essential to unlock their full capabilities and to ensure their successful integration into clinical applications.

## Highlights

• The **general strategies for designing EMNMs** are thoroughly summarized.
• The **recent, updated progress** in the clinical application of EMNMs toward the diagnosis and treatment are introduced.
• **Comparative analysis** of EMNMs across diverse systems, integrating variables is conducted.
• **Currentchallenges** faced by EMNMs for clinical translation, along with future possibilities and directions are discussed.

## Graphical abstract

**Figure:**
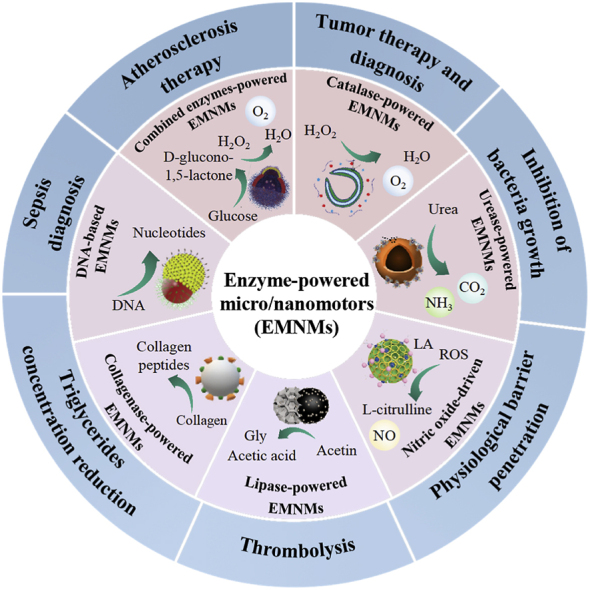

## 1 Introduction

Over the past decades, explosive growth has been witnessed in the research of micro/nanomotors, showing promising applications in the field of biomedicines. Among the various propulsion mechanisms developed, the enzyme-powered micro/nanomotors (EMNMs) are innovative due to their good biocompatibility and high selectivity. EMNMs involve anchoring enzymes on catalytic engines to generate self-propulsion effectively powering artificial micro/nanomotors [[1], [2], [3]]. EMNMs are rooted in biological systems, as almost all chemical reactions in the body are catalyzed by specific enzymes with high efficiency accompanied by the release of energy [2,4]. Moreover, the enzymatic reaction mediated by EMNMs can make use of harmful substances as fuels in living organisms, for instance, the decomposition of excessive H_2_O_2_ by catalase-based EMNMs in the tumor microenvironment and the removal of the dense extracellular matrix of solid tumors by collagenase-based EMNMs [[5], [6], [7]]. The enzymes of EMNMs themselves or their reaction products have various benefits for the human body, such as urease itself as an antibacterial agent, the anti-tumor and vascular endothelial repair activities of nitric oxide (NO), which bring unexpected therapeutic effects [[8], [9], [10]]. All of these represent innovative approaches to developing EMNMs for biomedical applications [[11], [12], [13], [14]]. Pioneering work was reported in 2005 by Mano and Heller, where glucose oxidase (GOx) and bilirubin oxidase (BOD) were attached to carbon fibers, resulting in a rapid self-propelling at the interface of water-O_2_ by the ion flow produced from an enzymatic reaction [12,15,16]. Since then, numerous systems of EMNMs have been developed, opening the door to subsequent application of EMNMs in biotechnological areas. EMNMs have been widely applied in drug delivery, biosensors, biological imaging, protein detection, cholesterol detection, and other aspects of biomedicine [[17], [18], [19], [20], [21]]. Compared with traditional micro/nanoparticles, EMNMs have obvious advantages in addressing biomedical challenges [[22], [23], [24]]. EMNMs integrate the excellent characteristics of nanomaterials, including large surface-to-volume ratio and surface activity, and the unique feature of directional chemotaxis in the presence of concentration gradients of their substrates [25], enabling highly efficient bio-separation and the targeted delivery of drugs or imaging agents [26,27]. Besides, EMNMs convert the surrounding energy into mechanical motion to propel themselves to penetrate specific complex biological environments and tissues more deeply and continuously, providing possibilities for overcoming the challenges in the diagnosis and treatment of many diseases, such as waste of drugs in surrounding tissues, penetrate physiological barriers, and adapt to changing physiological conditions, *etc*. [[28], [29], [30]]. In addition, EMNMs can be designed to accommodate the demands of a specific biological microenvironment by selecting appropriate materials and shapes with motivation powered by corresponding enzymes such as catalase [20], urease [31], nitric oxide synthase [21], collagenase [7], histamine-metabolizing enzyme [32] and so on. Switchable control over the motile behavior of EMNMs can be achieved by its engineering with various propelling forces designed to counterbalance each other [29,30]. Besides, the incorporation of multiple enzymes to power EMNMs can further enhance their mobility efficiency and expand the application scenarios in complicated microenvironments such as malignant tumors and diabetes [4].

**Fig. 1:**
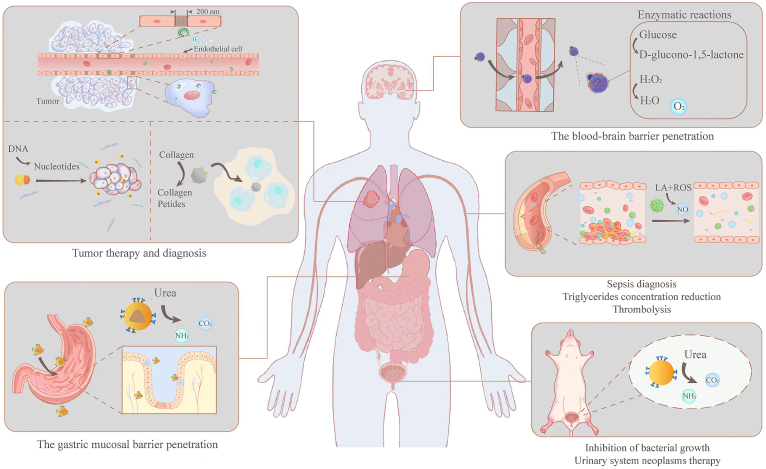
The driving methods and potential applications of EMNMs in clinical disease diagnosis and treatment.

This review provides an overview of the recent advances in the design and applications of EMNMs in disease diagnosis and treatment, with a particular focus on the specific problems that can be solved clinically by EMNMs through selecting proper compositions, structures, morphologies and the driving methods to penetrate biological barriers (Fig. 1). Bringing together the latest updates in the field, the properties of EMNMs driven by various enzymes are herein summarized into tables (Table 1, Table 2). Unlike existing literature, this review presents a unique comparative analysis of EMNMs across various systems based on factors such as materials, sizes, driving mechanisms, and environmental conditions, thus providing important insights into the current EMNMs research landscape.

**Table 1:** Summary of basic properties of single enzymatic reaction-propelled EMNMs.

Enzymes	Materials	Size (μm)	Concentration/Environment	Speed (μm/s)	Mechanism	Schematic diagram	Ref.
Catalase	Iron oxide particles	20–25	–	–	Bubbles propulsion		20
	Ultrasmall stomatocyte nanomotors (USSN)	≈0.15	2–20 mM	20.52 ± 0.35	Bubbles propulsion		6
	Polymer-based bottlebrush	≈0.1	2, 10 mM	15.5 or 23.6	Bubbles propulsion		5
	CAT-PDPA@ZIF-L micromotor particles	–	74.2mM–600mM	1590–5170	buoyancy regulation		33
Urease	Antibody-modified urease nanomotors (MSNP-Ur/PEG-Ab)	2	0, 12.5, 25, 50, 100, 200, and 300 mM	–	Phoretic propulsion		31
	Urease-functionalized polydopamine nanocapsule	1	0,50,100 mM, and real urine	4.56, 9.34,10.67	Phoretic propulsion		34
	Mesoporous silica nanoparticles (MSNPs)	≈0.41	25, 50, 100 mM	–	Phoretic propulsion		8
	Magnetic micro-propellers	1.5	20 mM	≈1.38	Phoretic propulsion		35
	Bio-inspired urease-powered poly-dopamine micromotor	≈2	5, 10, and 20 mM	0.78, 1.31,1.9	Phoretic propulsion		36
Nitric oxide synthase	MS/LA/RGD/UK	0.255	activated platelet inflammatory, endothelial cell	3.52,3.47	Self-diffusiophoresis		17
	hyperbranched polyamide/L-arginine (HLA)	0.12, 0.17, 0.385	5, 10, 20 wt%	3–13	Bubbles propulsion		21
	heparin-folate-cy5.5/L-arginine (HFCA)	0.04	–	–	Bubbles propulsion		37
	trehalose-L-arginine-phosphatidylserine (TAP)	0.2	1 μg/mL	4.5 or 5	Self-diffusiophoresis		38
	CD-LA-Au-aV	0.25	–	5–8	Bubbles propulsion		39
	poly-N-methacrylate-arginine (PMA)- triphenylphosphine (TPP)/paclitaxel (PTX)	0.31	–	3	Self-diffusiophoresis		–
Lipase	Lipase-modified mesoporous silica nanoparticles (LNMs)	≈0.43	1, 10, 100 mM triglyceride	–	Phoretic propulsion		40
	Multi-stimuli propelled Janus lipase-modified dendritic silica/carbon@Pt nanomotor (DMS/C@Pt)	≈0.98	1, 10 mM triglyceride	–	Phoretic propulsion		41
Collagenase	Collagenase-modified swimmers equipped with manganese ferrite nanoparticles (MF-NP)	0.03	ECM	–	Calcium gradient		7
DNase	PEG-Au-PAA/mSiO_2_ Janus nanoparticle	≈0.2	0, 0.5, 1, 2.5 μM	6.13, 7.71, 8.58, 9.35	Diffusiophoresis and the local thermal effect		42

**Table 2:** Summary of basic properties of combined enzymatic reaction-propelled EMNMs.

Enzymes	Materials	Size (μm)	Concentration/Environment	Speed (μm/s)	Mechanism	Schematic diagram	Ref.
GO_X_ and catalase	Carbon nanotubes	diameter 20–80 nm, length 0.5–5 mm	water	–	Bubble propulsion		43
	Sub-micron-sized Janus particles	0.8	400 mM glucose	–	Bubble propulsion		44
	Bowl-shaped nanocapsules	<5 nm	2.5 mM glucose	–	Bubble propulsion		45
	Asymmetric polymersome	–	1 M glucose	200	self-phoretic mechanism		46
	Cationic gold nanoclusters (CAuNCs@HA)	≈0.17	–	25.25 ± 0.33	Bubbles propulsion		47
	Metal-organic frameworks	≈0.041	1, 5, 25, 50 mM glucose	–	Bubbles propulsion		48
Catalase and urease	Streptavidin-functionalized polystyrene microspheres	0.79 μm	11.1 mM H_2_O_2_ and 213 mM urea	–	self-phoretic mechanism		49
GO_X_ and trypsin	Inorganic substance	800 nm	Water	0.72 μm/s and 0.1 μmol/L	Bubbles propulsion and phoretic propulsion		50
Urease and hyaluronidase	Janus nanomotors	≈0.093	5 mM urea, 0–0.8 nmol cm^−2^ in HA solutions		Bubbles propulsion		51

In addition to highlighting the latest progress and applications of EMNMs in disease diagnosis and treatment, we also dedicate a significant portion of this document to the discussion of challenges faced in this field, as well as the trends that are emerging toward its future development. Thus, this review serves as a unique resource to inform and promote the development of EMNMs toward more accurate diagnosis and treatment of clinical diseases, exploring new possibilities for overcoming severe challenges awaiting solutions in therapeutics.

## 2 Single enzymatic reaction-propelled EMNMs

Thanks to the various substrates for enzymatic reactions in the human body, the unique designs of single enzymatic reaction-propelled EMNMs hold immense promise to solve biomedical issues [22]. On the one hand, the energy converted by the enzymatic reactions endows the EMNMs with self-propulsion ability, realizing the deep penetration of various biological barriers, including the blood-urine barrier, blood-brain barrier, and blood-tumor barrier in an autonomous way [[22], [29], [52], [53], [54], [55]]. On the other hand, different types of single enzymatic reaction-propelled EMNMs catalyze specific reactions. For example, catalase-based EMNMs can consume excess H_2_O_2_ in the body, and nitric oxide-based EMNMs can produce NO to change the body environment, which can accommodate the medical applications in distinctive microenvironments [4]. For instance, the single enzymatic reaction-propelled EMNMs have been designed for targeted drug delivery with on-demand release and have been used as biosensors for diagnosis of disease, demonstrating their feasibility in both treatment and diagnosis. These enzymatic reactions-propelled EMNMs are listed in Table 1. In this section, we mainly introduced the composition of single enzymatic reaction-propelled EMNMs, design principles and their applications in diagnosis and treatment, including catalase-based EMNMs, urease-based EMNMs, nitric oxide-based EMNMs, collagenase-based EMNMs, DNase-based EMNMs and lipase-based EMNMs.

### 2.1 Catalase-based EMNMs

Conjugated with catalase into micro/nanomaterials, catalase-based EMNMs consume hydrogen peroxide (H_2_O_2_) to produce oxygen (O_2_) as a propulsion force. Over the past decades, catalase has been the most widely used enzyme to fuel micro/nanomotors [56]. Due to its good biocompatibility and excellent turnover rate, catalase-based EMNMs are widely used in environments with H_2_O_2_ [12,57,58], such as tumors [[59], [60], [61]] and sepsis sites [62]. The enrichment of H_2_O_2_ in the pathological region provides a possibility for catalase-based EMNMs to be used in the process of the diagnosis and treatment of these diseases. Catalase-based EMNMs are capable of realizing the rapid diagnosis of sepsis and deeper tissue penetration for tumor treatment.

**Fig. 2:**
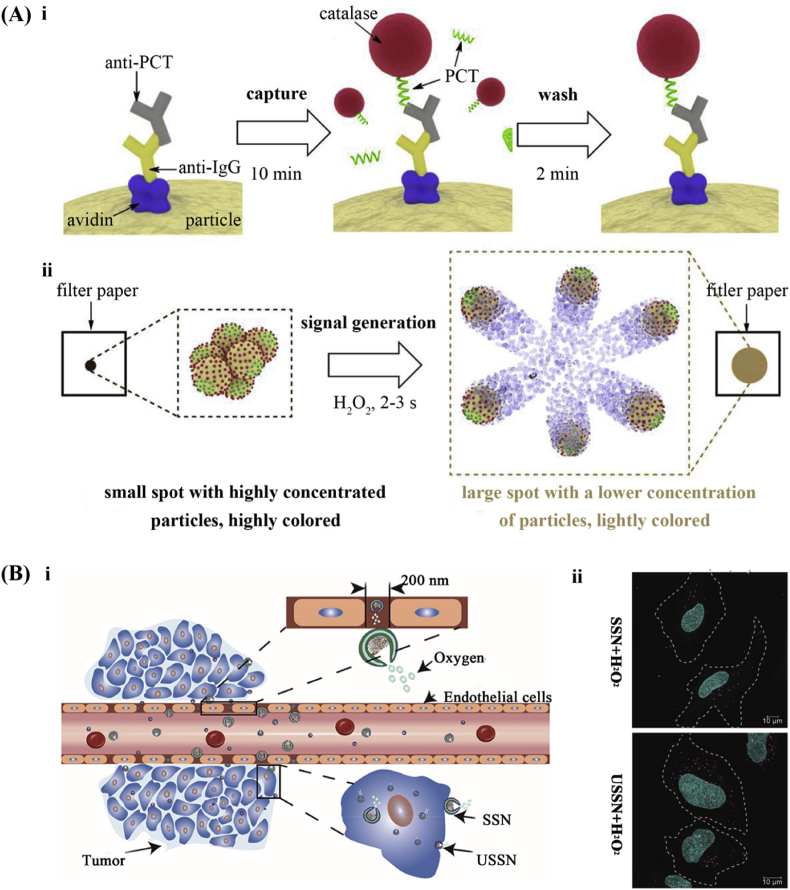
Representative examples of the designs for catalase-based EMNMs. (A) Self-propelled multifunctional Janus nanomotors: (ⅰ) the fabrication and (ⅱ) the mechanism of the self-propelled multifunctional Janus nanomotors. Reprinted with permission from ref [20]. Copyright 2019 Elsevier. (B) Ultra-small stomatocyte nanomotors: (ⅰ) the penetration process and (ⅱ) the cellular uptake by HeLa cells of the ultra-small stomatocyte nanomotors. Reprinted with permission from ref [6]. Copyright 2019 American Chemical Society.

Sepsis, a multiorgan dysfunction caused by an abnormal response to infection, influences 30 million people and results in the deaths of around 6 million patients annually. The rapid detection of procalcitonin (PCT), a biomarker of sepsis, can greatly increase the survival rate of patients with sepsis. To expedite detection, Russell et al. designed multifunctional Janus catalase-based EMNMs for measuring changes in nanoparticle motion colorimetrically to achieve the rapid detection of sepsis [20]. In this approach, iron oxide serves as the core material of the EMNMs, imparting both brown color and magnetic properties, reducing non-specific interference in biosensing applications and enhancing sensitivity for colorimetric analysis. During the reaction of the EMNMs, the PCT of a blood sample from sepsis patients would bind to the anti-PCT of the nanoparticles and it was then bound with catalase to form catalase-based EMNMs *via* immunoassay (Fig. 2A, ⅰ). The fabricated catalase-based EMNMs were dropped onto the filter paper, resulting in the appearance of a colored spot. Upon the addition of the catalase substrate, the nanomotors boned with catalase *via* an immunoassay, gained the ability of self-propulsion. This process produced larger, light-colored spots in a short time (Fig. 2A, ⅱ). This design enabled the detection of the biomarker of sepsis within 13 min, significantly reducing the diagnostic time as compared to the conventional laboratory assay for PCT which typically takes about 30 min. While various biosensors have demonstrated the ability to detect PCT at very low limits, they typically necessitate an incubation period of up to an hour and require prior separation of serum from the blood of the patient. In this sense, EMNMs show great potential for the rapid diagnosis of sepsis. Implementing rapid testing in the triage process enables the efficient prioritization of patients, facilitating timely access to life-saving interventions. Moreover, these micro/nanomotors could be easily converted to testing other blood biomarkers by simply replacing the antibodies in the Janus surfaces, broadening their application as biosensors.

In the field of tumor treatment, catalase-based EMNMs exhibit their unique merits in tumor inhibition and tissue penetration. To further enhance the ability of tissue penetration and cellular uptake for catalase-based small stomatocyte nanomotors (SSNs), Sun et al. designed ultrasmall stomatocyte nanomotors (USSNs) powered by catalase [6]. By incorporating PEG into a flexible polymer matrix, they achieved smaller vesicles with enhanced biocompatibility. The ultrasmall stomatocyte motor system enabled bubble propulsion at a concentration of 2 mM H_2_O_2_, with the velocity of nanomotors correlating to the O_2_ production. Compared to SSNs, the modification with PEG controlled the size of the catalase-based EMNMs and provided effective protection for the catalase. With this modification, USSNs demonstrated an improved ability of penetration, as they exerted a propulsive force across the mimic vasculature and the uptake of the USSNs by HeLa cells was increased (Fig. 2B, ⅰ). The USSNs showed higher cellular uptake efficiency and a 3.7 times larger fluorescence area was observed as compared to SSNs, by detecting the fluorescence from Nile Red encapsulated in EMNMs. In the presence of 2 mM H_2_O_2_, the fluorescence area of USSN was 2.2 times that of the SSN. This result indicated higher penetration of USSN into the tumor tissue (Fig. 2B, ⅱ). According to Li et al. work, molecular bottlebrush catalase-based EMNMs offered a unique platform for overcoming tissue penetration barriers [5]. The bottlebrush design coupled with the high activity of catalase enabled strong directional propulsion at an H_2_O_2_ concentration of 2 mM. Moreover, these molecular nanomotors exhibit superior stability in serum-containing cell medium and good biocompatibility in the blood. Furthermore, Gao et al. developed a new type of catalase-based EMNMs that can move at ultra-low hydrogen peroxide concentrations [63], based on which the centimeter-scale directional vertical motion was achieved through pH-regulated buoyancy control [33]. These aforementioned designs enable enhanced tissue penetration, while effectively promoting cancer treatment.

Although catalase has been extensively used in the design of EMNMs, it poses several challenges. For instance, the significant amount of gas it generates can lead to gas embolism, and H_2_O_2_ is a strong oxidizing agent that may cause toxicity to the human body [64]. As a result, researchers continue to explore alternative biofuels for EMNMs design.

### 2.2 Urease-based EMNMs

Urease, an oligomeric enzyme that contains nickel, catalyzes urea conversion to ammonia (NH_3_) and carbon dioxide (CO_2_). Urea concentrations in the human body generally range from 5 to 10 × 10⁻³ M, thereby establishing urease as a promising candidate for the development of micro/nanomotors [65]. In urease, there are specific amino groups capable of binding with the aldehyde groups and the phenolic hydroxyl groups which can act as reactive active sites to fabricate urease-based EMNMs. Urea is generally considered a metabolic waste product; however, it can be utilized by urease-based EMNMs, enabling new interesting functions.

**Fig. 3:**
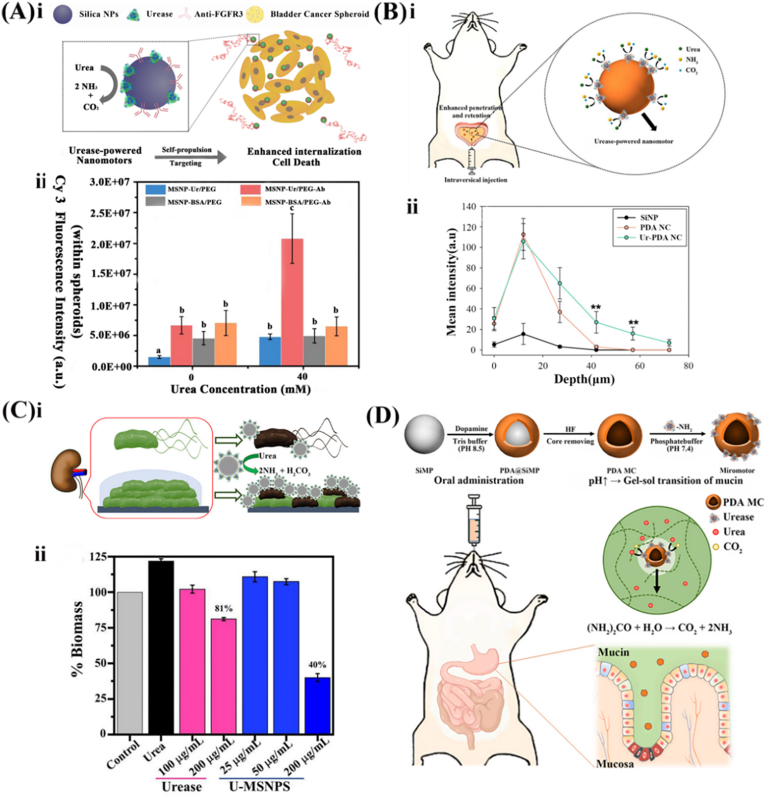
Representative examples of the designs for urease-based EMNMs. (A) Urease-based EMNMs based on mesoporous silica nanoparticles: (ⅰ) the fabrication of urease-based EMNMs based on mesoporous silica nanoparticles and (ⅱ) the quantification of the internalization of antibody-modified nanomotors into bladder cancer spheroids. Reprinted with permission from ref [31]. Copyright 2019 American Chemical Society. (B) Biocompatible and bioavailable urease-powered polymer nanomotors: (ⅰ) the structure and function of the nanomotors and (ⅱ) the mean fluorescence intensity of bladders with increasing depth after intravesical injection. Reprinted with permission from ref [34]. Copyright 2020 American Chemical Society. (C) Drug-free urease-based nanomotors: (ⅰ) the mechanism of the drug-free urease-based nanomotors and (ⅱ) the percentage of the biofilm biomass from uropathogenic E. coli remaining after treatment with U-MSNP nanomotors and excess urease. Reprinted with permission from ref [8]. Copyright 2021 American Chemical Society. (D) The mechanism of the bioinspired urease-based biopolymeric micromotor. Reprinted with permission from ref [36]. Copyright 2022 Elsevier.

The urease-based EMNMs produce persistent propulsion force to enhance the overall penetration efficiency of micro/nanomotors in treating urinary system neoplasms. Currently, the most common postoperative adjuvant therapy for bladder cancer after tumor resection is intravesical chemotherapy. However, this approach faces limitations due to the frequent act of urination, causing chemotherapeutic drugs to have a short retention time within the bladder. Consequently, this results in inadequate drug absorbance and limited contact time between the drug and the lesion. To improve the penetration efficiency within a given timeframe, an antibody-modified urease/polyethylene glycol mesoporous silica nanoparticles (MSNP-Ur/PEG-Ab) was developed to penetrate the human urinary bladder transitional papilloma RT4 cells' 3D spheroids [31] (Fig. 3A, ⅰ). By leveraging the urease-catalyzed decomposition of urea in urine, a persistent driving force was provided to realize the targeted delivery of anti-FGFR3 antibody in bladder cancer cells' 3D spheroids. The combination of the anti-FGFR3 enables the identification of bladder cancer cells while simultaneously inhibiting the proliferation of bladder cancer cell lines, ultimately leading to cellular apoptosis [[66], [67], [68]]. By integrating both targeting and motility features, the internalization efficiency was enhanced by 4 orders of magnitude (Fig. 3A, ⅱ). Besides, urease-functionalized polydopamine nanocapsules (Ur-PDA NC) were reported to improve penetration efficiency during intravesical chemotherapy. This was achieved through the physical adhesion effect of the PDA layer [69,70] in combination with the persistent propulsion force generated by urine decomposition [34] (Fig. 3B, ⅰ). The fluorescence intensity of Ur-PDA NC at the bladder wall's depth of 60 μm was observed nearly 20 times higher than that of PDA NC, indicating an exceptional penetration capacity of Ur-PDANC (Fig. 3B, ⅱ). In another publication, urease-based EMNMs synergistically combined with gold nanorods (AuNR) or magnetic continuum robots (MCRs) to enable drug-free therapy, offering more possibilities for the treatment of tumors [71,72].

In addition to providing the persistent propulsion force, urease-modified nanomotors generate HCO_3_^−^ and NH_3_, which neutralize acidic substances. This mechanism inhibits the growth of uropathogenic *E. coli* and the consumption of hydrochloric acid existing in the gastric mucosa. The weakly alkaline environment produced by NaHCO_3_ and NH_3_ is well-known for the ability to denature the structural protein of uropathogenic strains of *E. coli*. Vilela et al. reported urease-based mesoporous silica nanoparticles (U-MSNPS) for the uropathogenic *E. coli* associated infections [8] (Fig. 3C, ⅰ). Treatment with U-MSNPs led to a significant reduction in the biofilm's biomass, which reduced sharply to 40 % of the control group, thus showing a good antibacterial effect (Fig. 3C, ⅱ). Moreover, beyond their application in the urinary system, urease-based EMNMs can also decompose the urea in the stomach to facilitate the penetration of the gastric mucosal barrier. A recent report has highlighted the secretion of urease by *Helicobacter pylori.* as a defense against the severe acidic environment of the stomach and a means to decrease mucus viscosity by neutralizing the hydrochloric acid covering the gastric mucosa, facilitating *H. Pylory* propulsion [[73], [74], [75]]. Inspired by the strategy used by *H. pylori* to achieve penetration of mucin gels, Walker et al. designed an artificial system of reactive magnetic micro propellers that imitate this strategy to penetrate gastric mucin gels *via* surface-immobilized urease [35]. Similarly, Choi et al. reported a bioinspired urease-powered polydopamine (PDA) nanomotor as a biomimetic system of *H. pylori* for active oral drug delivery in the stomach [36] (Fig. 3D). During the reaction, urease catalyzes the hydrolysis of urea in the stomach to release NH_3_ and the increase of local pH value. This elevation in pH includes a gel-sol transition in the gastric mucus layer, thereby reducing mucus viscosity and facilitating the penetration of the nanomotor into the stomach wall through propulsion.

Moreover, urease-based EMNMs can also achieve targeted treatment in other digestive diseases, such as intestines. Hortelão et al. designed a nanovesicle with urease modified inside [76], which simulates oral medication and is engineered to withstand the acidic environment, realizing targeted delivery. Urease is encapsulated within the inner compartment of the liposome known as LipoBots-Inside (LB-I), where the lipid bilayer protects it from degradation in the stomach. Upon reaching the intestine, the urease is released by the stimulation of bile salt, facilitating propulsion. The results demonstrated that these nanovesicles retained their self-propulsion ability under acidic conditions, effectively protecting urease from denaturation and advancing the development of novel active drug delivery systems.

### 2.3 Nitric oxide-based EMNMs

The endogenous biochemical reactions existing within the human body offer valuable insights for the design of micro/nanomotors. It is widely known that nitric oxide synthase (NOS) and endogenous reactive oxygen species (ROS) are produced in large quantities by inflammation reactions and postinjury stress [77], which can convert L-arginine (LA) into L-citrulline and nitric oxide (NO). The carboxyl or amino groups in arginine can serve as reactive sites, attracted to biomaterials. Inspired by this, a movement system has been designed in which LA is utilized as fuel and fixed on the MNMs [21,[78], [79], [80]]. Importantly, in this reaction, both the reactant and products are beneficial to the human body [77,78].

**Fig. 4:**
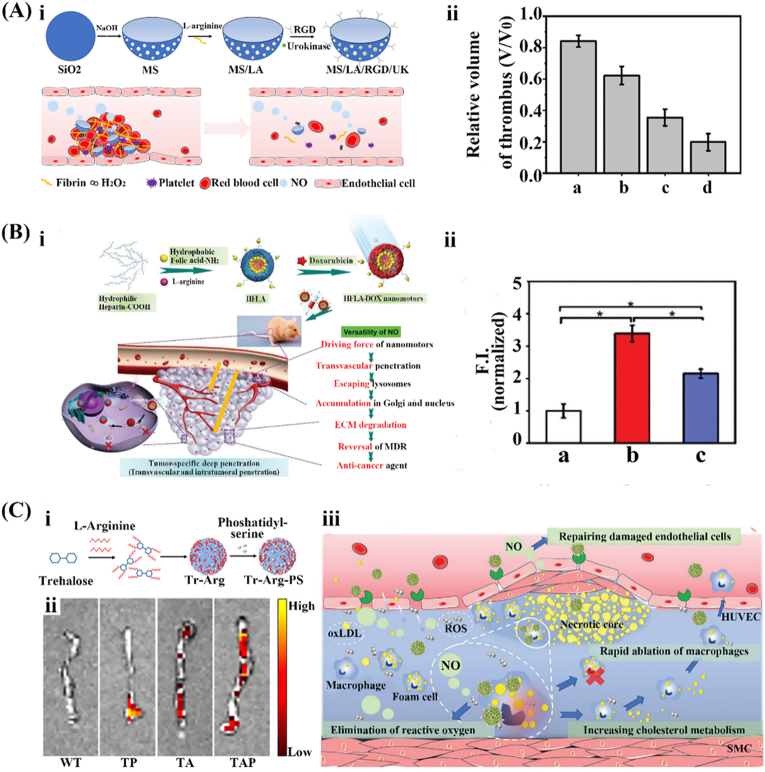
Representative examples of the designs for NO-based EMNMs. (A) NO-driven nanomotors: (ⅰ) the fabrication of nanomotors with bowl-shaped mesoporous silica and (ⅱ) corresponding thrombus relative volumes at 0 and 7 d: (a) PBS, (b) UK, (c) non-nanomotors, (d) nanomotors. Reprinted with permission from ref [17]. Copyright 2021 Elsevier. (B) (ⅰ) The fabrication process of HFLA-DOX nanomotors and (ⅱ) corresponding fluorescence intensity (samples for a: cy5.5 labeled HF-DOX, b: cy5.5 labeled HFLA-DOX, c: cy5.5 labeled HFLA-DOX + uric acid). Reprinted with permission from ref [84]. Copyright 2020 Wiley. (C) (ⅰ) Carrier-free trehalose-based nanomotors and (ⅱ) nanomotors with dual-mode propulsion preparation and (iii) the mechanism of their application in the treatment of atherosclerosis. Reprinted with permission from ref [38]. Copyright 2022 American Chemical Society.

NO-driven micro/nanomotors have attracted much attention in the field of synergetic thrombolysis to prevent the recurrence of thrombosis due to their enduring movement ability and the function of repairing damaged blood vessels. At present, drug treatment remains the primary choice for clinical therapy of thrombosis [81]. However, a low drug retention rate at the thrombus site, caused by excessive blood flow rate, hampers the thrombolytic effect and increases the risk of recurrence [82,83]. To address this challenge, a bowl-shaped mesoporous silica nanomotor (MS) driven by NO was designed, which was modified by arginine-glycine-aspartic acid (RGD) polypeptide and simultaneously loaded with LA and urokinase (UK) [17] (Fig. 4A, ⅰ). LA can react with excessive ROS in the thrombus microenvironment to generate NO, while UK is a thrombolytic drug. The MS/LA/RGD/UK nanomotors possess the combined advantages of RGD's thrombus surface targetability and the motility of the nanomotors, facilitating continuous penetration and retention of the UK at thrombus sites. Consequently, the thrombolytic volume of the nanomotors group was 2 times higher than that of thrombolytic drugs alone (UK group) after 7 d of treatment, demonstrating the excellent thrombolytic ability of the nanomotors (Fig. 4A, ⅱ). In this design, ROS consumption and NO generation work together to enable auxiliary therapeutic effects, while NO gas produced also provides propulsion. Furthermore, the MS/LA/RGD/UK nanomotors mitigated the oxidative stress in inflammatory endothelial cells by consuming ROS. Moreover, the produced NO had the physiological functions of anti-platelet adhesion and promotion of endothelial cell growth, effectively preventing the recurrence of thrombus.

The remarkable motion ability of NO-driven micro/nanomotors and the biomedical functions of the produced NO synergistically enhance the deep penetration of drugs in tumor tissues too, thereby improving the anticancer drug effect. Poor drug permeability and multidrug resistance (MDR) in solid tumors are the two major hurdles contributing to the failure of current chemotherapy, immunotherapy, and other cancer treatments [37,84]. This is particularly critical in cancer due to the increases in solid stress and extracellular matrix modifications which render the tissue more compact, stiff, and thus harder to disrupt [85]. To achieve deep drug penetration while effectively reversing MDR in cancer chemotherapy, a zero-waste nanomotor loaded with heparin/folic acid/l-arginine (HFLA-DOX) was designed [84] (Fig. 4B, ⅰ). The HFLA-DOX nanomotors utilized the NO generated by the reaction of LA with a high concentration of NOS and ROS in the tumor environment to propel themselves and the loaded drugs deep into the tumor tissue. Moreover, the produced NO promoted the normalization of the tumor vascular system and the degradation of the extracellular matrix (ECM), facilitating the enhanced penetration of nanomotors into the tumor tissue while reversing the MDR of cancer cells. The fluorescence intensity of the HFLA-DOX group was approximately three times higher than that of the HF-DOX group, demonstrating superior penetration ability (Fig. 4B, ⅱ).

Recently, NO-driven micro/nanomotors have demonstrated their distinctive advantages in targeting and synergistic treatment of atherosclerosis (AS). In the current treatment for AS, the targeting and penetration of drugs are still insufficient, requiring new therapeutic tools and delivery systems to address AS [86]. A new type of NO-driven and carrier-free nanomotor based on the reaction between trehalose (Tr), L-arginine (Arg), and phosphatidylserine (PS) was reported [38] by Wu et al. (Fig. 4C, ⅰ). The high concentration of ROS and NOS within the plaque microenvironment of AS allowed for the chemotactic behavior of Tr-Arg-PS (TAP) nanomotors when they react with L-arginine enabling the first-step targeting to the AS site. Subsequently, a coating of PS enabled precise targeting of macrophages within the AS plaque, representing the second-step targeting. Imaging results demonstrated that the TAP nanomotors group exhibited 4.6 times higher efficiency compared to the TP NPs (Fig. 4C, ⅱ), thus confirming their targeting capability and effectiveness against AS. Compared with traditional functional nanocarriers, this carrier-free system greatly enhances the drug loading efficiency, showing the potential of overcoming the challenges of carrier degradation and incomplete drug release. Similarly, another type of nanomotor was reported for the treatment of AS. This nanomotor was composed of β-cyclodextrin and LA, covalently bound and self-assembled with immobilized Au nanoparticles. Different from system of TAP, which operates through the autophagy of macrophages, LA offers greater flexibility and can be applied to a wider range of scenarios. In this way, the nanomotor achieved multi-pathway microenvironment regulation in the treatment of AS [39]. Notably, this nanomotor utilized NO and near-infrared light for dual actuation to enhance targeting and penetration (Fig. 4C–iii).

### 2.4 Collagenase-based EMNMs

Collagenase is an enzyme that can cleave the peptidic bond between Hydroxyproline (Yaa) and the subsequent Glycine (Gly) in Gly-Xaa-Yaa (Glycine-Proline-Hydroxyproline) repeats of the collagen in the presence of calcium as a co-factor, leading to the breakdown of collagen [87]. Collagen, the substrate for collagenase, is widely found in ECM, and its overexpression is associated with tumor growth and metastasis [88,89]. Collagenase is negatively charged and can be bound to positively charged biomaterials by electrostatic interactions [7]. In addition, collagenase can form stable bonds with biomaterials containing maleimide groups through “click” chemistry after being thiolated [90]. Based on these characteristics, collagenase was immobilized on MNMs to develop collagenase-powered motors for targeted degradation of collagen [88]. Unlike most EMNMs, collagenase-based EMNMs do not rely on bubble propulsion; instead, they enhance tissue penetration through degradation reactions and may facilitate the efficient diffusion of other drugs.

**Fig. 5:**
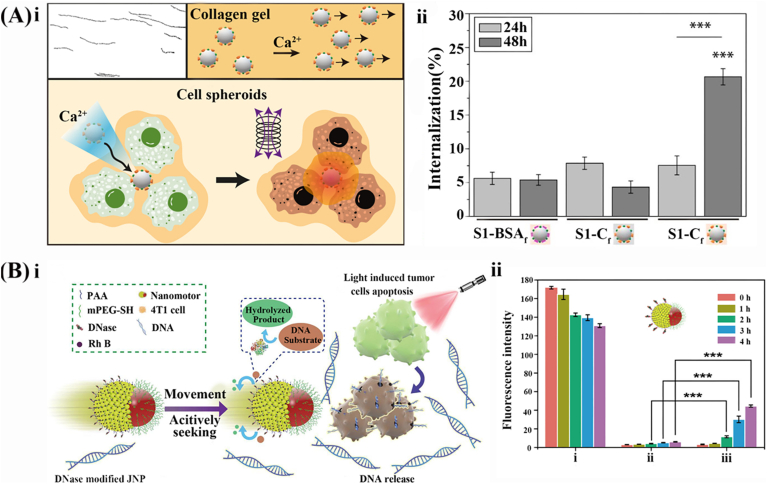
Representative examples of the designs for collagenase-powered EMNMs and DNA-based EMNMs. (A) Collagenase-powered nanomotors: (i) the mechanism of the self-propelled micro-nanomotors powered by collagenase and equipped with superparamagnetic nanomaterials and (ii) comparison of the penetration ability of different nanoparticles. Reprinted with permission from ref [7]. Copyright 2020 American Chemical Society. (B) DNase functionalized Janus nanoparticle: (ⅰ) the mechanism of DNase functionalized Janus nanomotors and (ⅱ) the fluorescence intensity of apoptotic tumor cells and normal cells of the DNase functionalized Janus nanomotors. Reprinted with permission from ref [42]. Copyright 2021 American Chemical Society.

The collagenase-powered micro/nanomotors can decompose excessive collagen in the ECM of the tumor cell microenvironment, thus enhancing tissue penetration for the delivery of anticancer drugs or imaging agents. Certain solid tumors such as breast, pancreatic, colorectal, ovarian, and lung cancer, exhibit a dense ECM with higher collagen content which is associated with a poor prognosis [88]. A type of bio-inorganic micro-swimmers based on polystyrene (PS) particles was reported, modified with collagenase to use collagen as fuel and calcium as a trigger for the movement. These micro-swimmers also incorporated superparamagnetic manganese ferrite nanoparticles for heat delivery, potentially enabling synergistic medical interventions such as hyperthermia [7] (Fig. 5A, ⅰ). In the presence of calcium, collagenase harnessed collagen from the ECM in the tumor cell microenvironment as the substrate to provide a continuous driving force for nanomotors. This enabled the nanomotors to penetrate deeply into a 3D cell spheroid of the human primary osteosarcoma cell line (SaOS-2 cell). Experimental results demonstrated that the internalization rate of the nanomotors group was 15 % higher than that of the non-nanomotors group, thus confirming the superior penetration capability of nanomotors (Fig. 5A, ⅱ).

### 2.5 DNase-based EMNMs

Most DNA-based EMNMs utilize enzymes with a high affinity to nucleic acid such as DNase [1]. DNase is a group of glycoprotein endonucleases capable of catalyzing the hydrolytic cleavage of phosphodiester linkages in a DNA backbone. The amino group in DNase can bind to the carboxyl group on the biomaterials, enabling the fabrication of DNase-driven EMNMs that exhibit unique chemotactic behavior, moving towards regions of higher DNA concentration.

EMNMs functionalized with DNase exhibit excellent chemotaxis along the concentration gradient of DNA released from the senescent and apoptotic cells to realize directional motion toward tumor cells [91]. It has been reported that cell-free tumor DNA released from apoptotic or dying tumor cells serves as a biomarker for early diagnosis, prognosis, and monitoring [[92], [93], [94], [95], [96]]. Compared to other methods for detecting circulating tumor cells (CTCs), the detection of DNA through the DNase-based EMNMs offers greater sensitivity and may enable personalized disease analysis, including the assessment of genetic and epigenetic aberrations [97]. Building upon this, DNase-functionalized Janus nanoparticles (JNP) powered by ultralow levels of DNA ranging from nanometer to micron were designed [42](Fig. 5B, ⅰ). These JNP sensed DNA signals released by the apoptotic tumor cells, leading to their movement towards tumor cells with enhanced fluorescence intensity (Fig. 5B, ⅱ). This design holds promise for detecting diseases associated with apoptotic cells by leveraging quantitative knowledge of DNA levels.

### 2.6 Lipase-based EMNMs

Lipase, a triacylglycerol acyl hydrolase, catalyzes the conversion of triglycerides to glycerin and fatty acids at a rapid rate, specifically at the oil-water interface. Lipase-based EMNMs are fabricated *via* electrostatic adsorption and aldehyde group anchoring in biomaterials. The movement of these EMNMs relies on the gradient of products generated by the degradation of triglyceride molecules [[40], [41], [98], [99]]. The presence of lipase endows a dual function for MNMs, realizing the clearance of triglycerides while providing continuous propulsion for the MNMs. Efforts have been made to achieve rapid clearance of abnormally accumulated triglycerides [40]. Recently, lipase-powered mesoporous silica micro/nanoparticles have been reported to exhibit the ability to enhance Brownian motion for an extended period while efficiently degrading triglycerides [40]. Also, it is worth noting that hyperlipidemia is commonly associated with elevated plasma triglyceride levels. Therefore, the rapid clearance of plasma triglycerides can alleviate symptoms related to hyperlipidemia. Thus, lipase-based EMNMs hold tremendous potential in addressing diseases relevant to hyperlipidemia, including atherosclerosis and acute pancreatitis.

## 3 Combined enzymatic reaction-propelled EMNMs

To diversify the movement environment and broaden their application range, EMNMs conjugated with multiple enzymes have been developed. These EMNMs utilize combined enzymatic reactions, employing at least two enzymatic reactions for self-propulsion, namely cascade enzymatic reactions or multiple enzymatic reactions. Compared with the single enzymatic reaction-propelled EMNMs system, the combined enzymatic reaction-propelled systems could effectively accelerate movement and enhance control capabilities, thereby broadening the range of potential applications for EMNMs and further validating the feasibility of EMNMs for treatment and diagnosis [4]. Table 2 provides a comprehensive summary of various systems utilizing combined enzymatic reactions-propelled EMNMs.

**Fig. 6:**
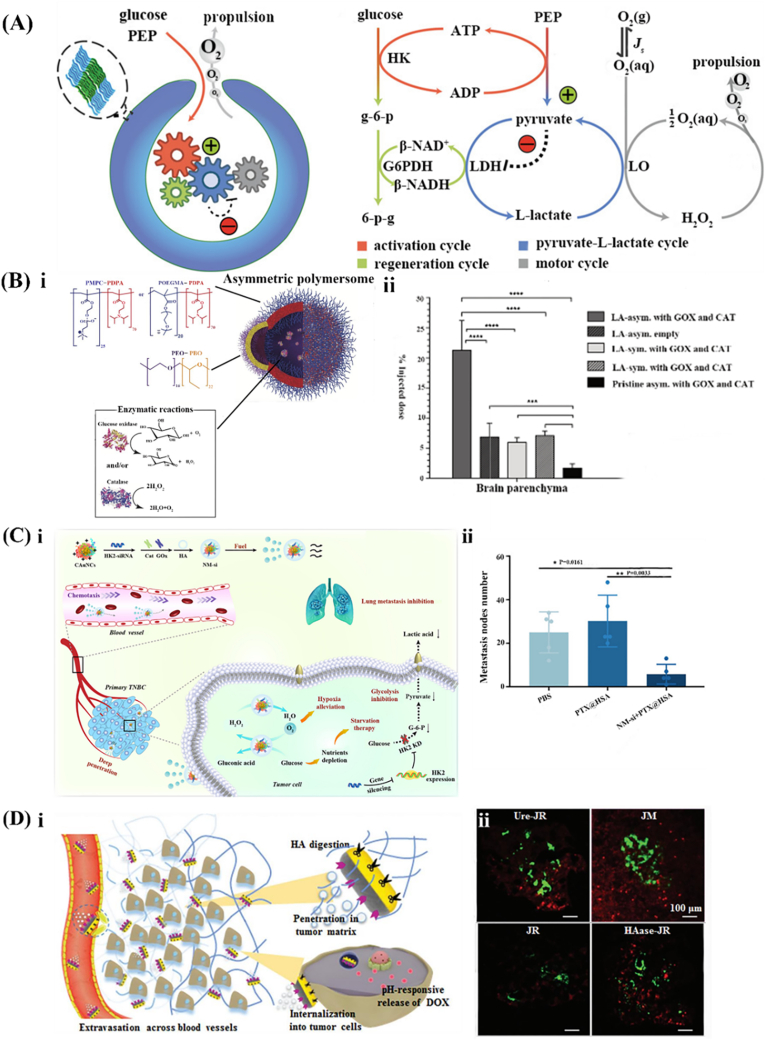
Representative examples of the designs for combined enzyme-powered EMNMs. (A) The design of the compartmentalized metabolic network. Reprinted with permission from ref [45]. Copyright 2016 American Chemical Society. (B) Autonomous nanoscopic swimmer: (ⅰ) the structure of the autonomous nanoscopic swimmer and (ⅱ) the percentage of the injected dose found in the rat brain parenchyma (C) Self-propelled nanomotor with HK-2 siRNA (NM-si): (ⅰ) the mechanism of NM-si and (ⅱ) the number of metastasis nodules. Reprinted with permission from ref [47]. Copyright 2021 Elsevier. (D) Icebreaker-inspired Janus nanomotors: (ⅰ) the mechanism of the icebreaker-inspired Janus nanomotors and (ⅱ) the CLSM images of DOX distribution (red) and CD31 of vascular endothelial (green) in tumor tissues. Reprinted with permission from ref [51]. Copyright 2021 The Royal Society of Chemistry.

Glucose oxidase (GO_X_) and catalase (CAT) are combined with biomaterials to create GO_X_/CAT system-propelled EMNMs. These EMNMs utilize cascade enzymatic reactions to achieve self-propulsion. In this process, glucose is initially decomposed into D-glucono-1,5-lactone and H_2_O_2_. Subsequently, the fast degradation of H_2_O_2_ into H_2_O and O_2_ by catalase generates driving forces [12]. While the reaction catalyzed by GOx is an oxygen-consuming system, the reaction catalyzed by CAT produces oxygen. This interplay allows the EMNMs to stabilize the gaseous environment, ensuring that the generated gas does not interfere with detection accuracy [100]. This cascade enzymatic reaction endowed MNMs higher speeds *via* combining multiple propulsion mechanisms and adapting to various environments, to realize applications such as drug delivery and tissue uptake. As reported by Pantarotto et al., the GO_X_/CAT system was modified on carbon nanotubes, successfully achieving the nanotubes’ propulsion [43]. Building on this foundation, sub-micron-sized Janus particles with enhanced diffusion in the presence of glucose were developed [44]. Additionally, to explore the applications of GO_X_/CAT system-propelled EMNMs in molecular lifelike systems, Nijemeisland et al. designed bowl-shaped nanocapsules with tunable and persistent performance under out-of-equilibrium conditions [45] (Fig. 6A). In this system glucose was converted into O_2_ to provide propulsion force, showing the potential of developing GO_X_/CAT system-propelled EMNMs for biological transformation.

Based on the research mentioned above, the GO_X_/CAT system-propelled EMNMs are increasingly employed in solving clinical problems. For the diseases of the central nervous system, a targeted approach was developed using low-density lipoprotein receptor-related protein 1 (LRP-1) targeting peptide Angiopep-2 modified asymmetric polymersome (LA-asym) with GO_X_ and CAT to penetrate the blood-brain barrier [46] (Fig. 6B, ⅰ). Through the synergistic catalytic activity of GO_X_ and CAT, significant propulsion was achieved to penetrate the blood-brain barrier. Notably, compared with the non-chemotactic polymersome control group, LA-asym with GOX and CAT exhibited approximately four times greater delivery into the rat brain parenchyma (Fig. 6B, ⅱ). Moreover, in the field of highly metastatic malignant tumors, the enzymatic decomposition of glucose by GOX effectively reduces glucose levels and increases oxygen concentration in the tumor, thus disrupting their energy supply and ameliorating hypoxic microenvironment. Simultaneously, CAT mitigates the toxic effects of H_2_O_2_ produced by GOX, protecting the human body from potential harm. Yu et al. reported a new type of EMNMs that embedded the GO_X_/CAT system in the cationic gold nanoclusters (CAuNCs@HA) with hexokinase-2 siRNA to create a self-propelled nanomotor (NM-si). This nanomotor was demonstrated with the capability of boosting hypoxia alleviation, inhibiting glycolysis, and promoting anti-metastasis [47] (Fig. 6C, ⅰ). Compared with control groups, the group treated with NM-si displayed the lowest number of metastasis nodes (Fig. 6C, ⅱ). Similarly, in another study, UCNPs/TAPP@ZIF-8@Catalase/GO_X_ (UTZCG) was fabricated using zeolitic imidazolate framework-8 (ZIF-8) to encapsulate up-conversion nanoparticles (UCNPs) and 5,10,15,20-tetrakis (4-aminophenyl) porphyrin (TAPP) [48]. The GO_X_ played a vital role in digesting glucose to starve the tumor cells, simultaneously generating singlet O_2_ from the H_2_O_2_, which propelled nanomotors and promoted photodynamic therapy triggered by TAPP.

In contrast with the cascade enzymatic reactions-propelled EMNMs, the multiple enzymatic reactions-propelled EMNMs capitalize different catalytic reactions occurring simultaneously, which lead to an enhanced self-diffusion and an increased speed of movement. For instance, Dey et al. designed a nanomotor functionalized with urease and catalase. They observed that its diffusion exhibits a stronger dependence on the substrate concentration, demonstrating that the motion of the nanomotor could be conveniently controlled by changing the substrate concentration [49]. Similarly, Schattling et al. reported Janus MNMs modified with GO_X_-Pt and trypsin which showed a significant increase in the speed of diffusion in the presence of glucose and peptides [50]. Noteworthy, compared with commonly used gold nanoparticles, platinum nanoparticles exhibit superior catalytic properties in hydrogenation and oxidation reactions. However, they are associated with poor biocompatibility and may induce biological toxicity. In another context, overcoming various biological and pathological barriers remains a challenge for cancer chemotherapy. The concept of icebreakers offers a new idea - these typically rely on the force of propellers to navigate and the gravity of the bow to split the ice. Inspired by this mechanism, a Janus nanomotor (JM) was designed to modulate the tumor microenvironment by degrading the ECM [51](Fig. 6D, ⅰ). In the tumor microenvironment, a high concentration of hyaluronic acid has strong viscosity, greatly limiting the diffusion of drugs. To address this, the hyaluronidase and urease were fixed on both sides of JMs, to provide a driving force from one side and clear the hyaluronic acid for penetration of tumor ECM on the other side. The CLSM image demonstrated a significantly stronger fluorescence intensity of DOX (red) in tumor mass indicating an enhanced cellular uptake after JMs administration (Fig. 6D, ⅱ).

## 4 Conclusions and perspectives

Over the past few decades, great efforts have been devoted to developing EMNMs that excel in drug delivery, tissue penetration, and biomedical sensing. Aiming to offer a thorough understanding of the classification and potential applications of EMNMs, this review summarizes the fundamental properties of various enzyme-driven EMNMs, complemented by clear comparative analysis in tables. Particular emphasis is given to their significance in the treatment and diagnosis of clinical diseases, delving into the latest advancements in EMNM research.

Thereinto, EMNMs have exhibited immense potential for their widespread usage in diseases caused by abnormal enzyme substrate accumulation or those occurring in an environment containing said enzymatic substrates. For anticancer research, especially for malignant tumors with high metastatic potential, GOX-based EMNMs can achieve starvation therapy to tumors and the collagenase-based EMNMs can decompose collagen in the tumor cell microenvironment, thus enhancing tissue penetration. Moreover, EMNMs leverage the unique effect of enzymes on tumor cells to enable magnetodynamic or photodynamic synergistic therapy by carrying specialized particles to achieve an efficient treatment strategy. In addition, EMNMs can effectively eliminate substances harmful to the human body. Another crucial aspect of EMNMs lies in their diagnostic capabilities, particularly demonstrated by DNase-based EMNMs, which can accurately detect the location of tumor cells through minimal changes in DNA concentration. Notably, as a drug carrier, EMNMs have been proven to overcome various drug transport barriers such as the blood-brain barrier and blood-tumor barrier.

However, the application of these EMNMs in the treatment of clinical diseases is not yet fully achieved. A few considerable gaps remain to be overcome before EMNMs can be widely used as therapeutics for clinical diseases. Firstly, the stability and durability of EMNMs require improvement, since the enzyme activity is vulnerable to the pH and temperature in the human tissues, with a high risk of deactivation before reaching the targeted sites. Additionally, most EMNMs rely on bubble propulsion and self-phoretic mechanisms, which complicate the precise control of their motion in the human body. Addressing these issues involves thoroughly investigating the interactions between EMNMs and biological systems. Comprehensive safety assessments of their potential toxicity and immunogenicity are prerequisites, with special emphasis on biodegradability and evasion of immune clearance in blood vessels. Current studies often lack long-term assessments, which are critical for determining safety in clinical applications. Transient self-destroyed micromotors in recent works have demonstrated promising control over degradation on demand, which could be adopted in the future design of EMNMs [101,102]. Furthermore, large-scale production of EMNMs with guaranteed quality remains a significant challenge. The high cost of enzyme extraction and purification, difficulties in achieving precise fabrication, and variability in complex biological environments all hinder the clinical transformation of EMNMs. Presently, most EMNMs research is still at the stage of *in vitro* experimental studies, highlighting the considerable distance to be covered before real applications.

Despite these limitations, EMNMs hold immense potential for the future development of cutting-edge diagnostics and therapeutics. There are several strategies that can be explored to enhance their efficiency, stability, and durability. Firstly, the development of more efficient and sophisticated enzymes—either engineered (nanozymes) or natural—will be critical for enhancing the performance of EMNMs, particularly those enzymes that are more relevant to specific diseases and more selective to pathological environments. Currently, most EMNMs rely on fuels in high concentration, such as glucose, H_2_O_2_ and urea, typically in mM or even M level. Developing enzymes capable of operating efficiently at lower substrate concentrations would enable EMNMs to function more effectively within physiological and pathological environments. Additionally, EMNMs that utilize disease-associated enzymes or substrates could offer targeted diagnostic and therapeutic potential, such as alkaline phosphatase, which is often associated with liver diseases (hepatitis, cirrhosis) or bone diseases (fractures, osteoporosis), but no alkaline phosphatase-driven EMNMs have yet to be developed. Meanwhile, advancements in the material design of carriers and the incorporation of more efficient signal transduction mechanisms could significantly improve the overall performance of EMNMs. For instance, based on the fact that magnetic nanomotors typically exhibit precise movement when controlled by a magnetic field [103], integrating magnetic carriers into EMNMs could further enhance their controllability. In the other field, by employing bionic concepts, EMNMs could be integrated with human cells, such as encapsulating EMNMs in erythrocytes or platelet membranes, creating living robots [104]. This integration could offer a feasible solution to evade the immune system, but more efforts have to be devoted to achieving stable integration. Besides, incorporating contemporary detecting methods such as ultrasound (US), photoacoustic (PA) or magnetic resonance imaging (MRI) with EMNMs can open new possibilities, enabling deep tissue imaging, real-time monitoring, and disease process visualization in living organisms. A step-by-step approach, starting with *in vitro* diagnosis and moving toward *in vivo* treatment, would be considered an ideal route for future research to promote their industrialization and final clinical application.

In summary, the EMNMs possess unique merits, making them highly promising for various applications such as drug delivery, penetration, biosensing, *etc*., while still facing some challenges concerning efficiency, stability, and biocompatibility. Therefore, further research and exploration are essential to unleash their full potential and ensure successful integration into clinical applications. By summarizing recent works in this field, identifying the current gaps and outlining future solutions, this review aims to accelerate breakthroughs of EMNMs for the achievement of solving clinical problems in a real sense - while influencing various domains of grand medical challenges and novel technology.

## CRediT authorship contribution statement

**Jinpeng Zhao:** Writing – review & editing, Writing – original draft, Software, Resources, Investigation, Funding acquisition, Formal analysis, Data curation, Conceptualization. **Banghui Wang:** Validation, Software, Methodology, Formal analysis, Data curation, Conceptualization. **Mingzhe Yan:** Writing – original draft, Visualization, Validation, Software, Project administration, Data curation, Conceptualization. **Yuxin Liu:** Writing – original draft, Validation, Investigation, Formal analysis, Data curation, Conceptualization. **Ruizhe Zhao:** Writing – original draft, Validation, Supervision, Resources, Project administration, Methodology, Data curation. **Xuezhe Wang:** Software, Methodology, Investigation, Formal analysis, Data curation, Conceptualization. **Tianyi Shao:** Writing – original draft, Methodology, Investigation, Data curation, Conceptualization. **Yifei Li:** Writing – original draft, Project administration, Methodology, Formal analysis, Data curation, Conceptualization. **Muhammad Imran:** Validation, Formal analysis, Data curation, Conceptualization. **Mingze Ji:** Methodology, Investigation. **Hong Zhao:** Conceptualization, Data curation, Investigation, Writing – review & editing. **Carlos F. Guimarães:** Writing – review & editing, Supervision, Resources, Funding acquisition, Data curation, Conceptualization. **Guotai Li:** Writing – review & editing, Software, Funding acquisition, Formal analysis, Conceptualization. **Qihui Zhou:** Writing – review & editing, Writing – original draft, Investigation, Funding acquisition, Formal analysis, Data curation. **Rui L. Reis:** Writing – review & editing, Software, Resources, Funding acquisition, Conceptualization.

## Ethics approval and consents to participant

No ethics are included in this manuscript.

## Declaration of competing interest

Rui L. Reis is an associate editor for Bioactive Materials and was not involved in the editorial review or the decision to publish this article. There is no conflict of interests involved in this study.
